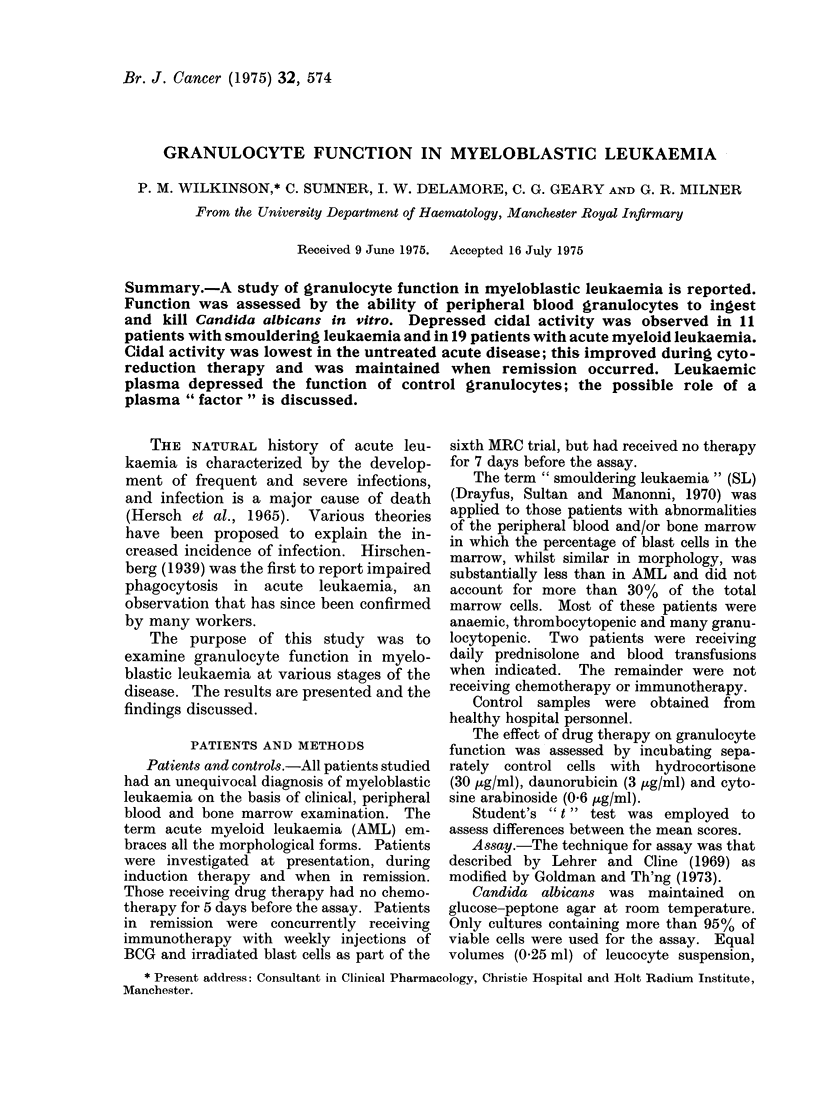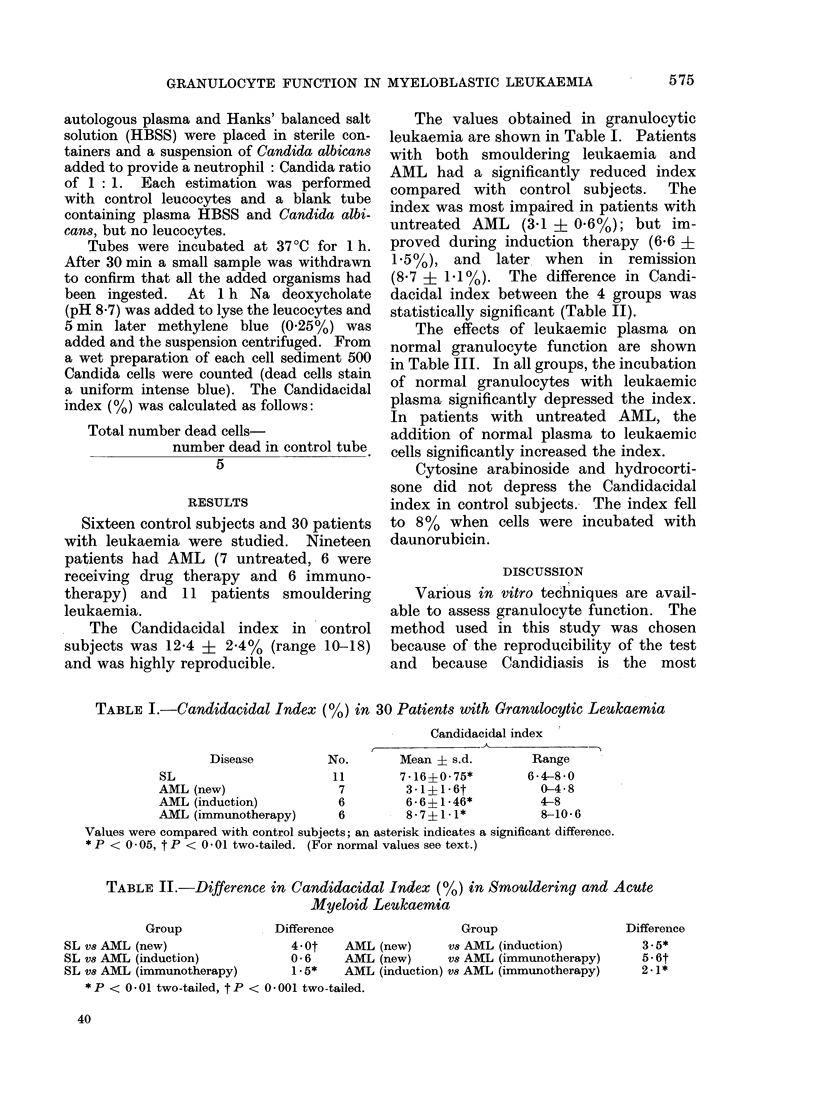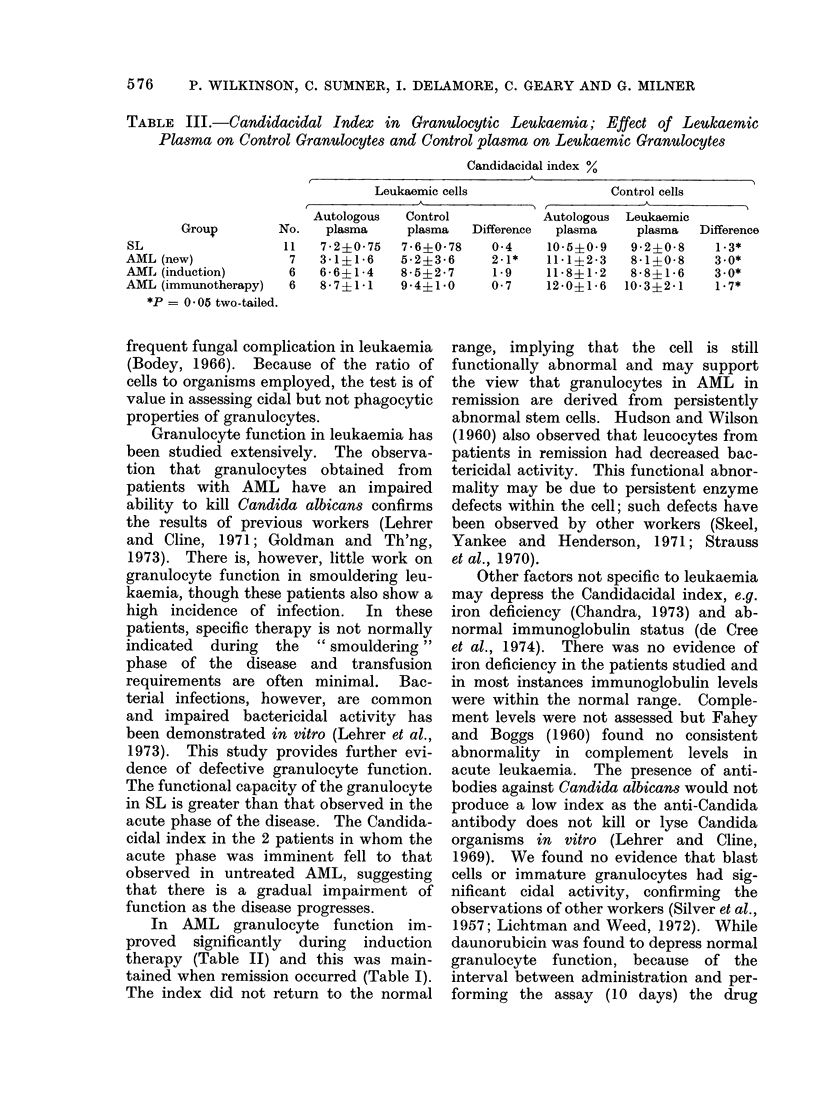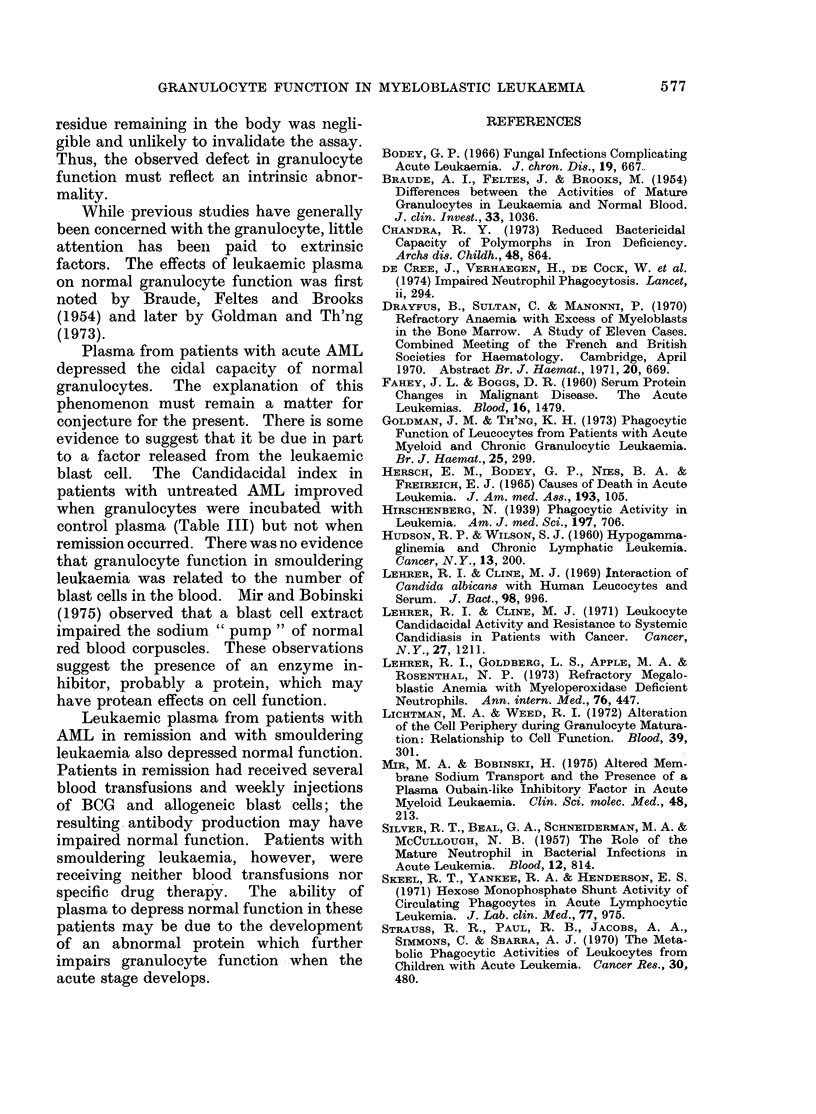# Granulocyte function in myeloblastic leukaemia.

**DOI:** 10.1038/bjc.1975.263

**Published:** 1975-11

**Authors:** P. M. Wilkinson, C. Sumner, I. W. Delamore, C. G. Geary, G. R. Milner

## Abstract

A study of granulocyte function in myeloblastic leukaemia is reported. Function was assessed by the ability of peripheral blood granulocytes to ingest and kill Candida albicans in bitro. Depressed cidal activity was observed in 11 patients with smouldering leukaemia and in 19 patients with acute myeloid leukaemia. Cidal activity was lowest in the untreated acute disease; this improved during cytoreduction therapy and was maintained when remission occurred. Leukaemic plasma depressed the function of control granulocytes; the possible role of a plasma "factor" is discussed.


*Br. J. Cancer* (1975) **32**, 574

### GRANULOCYTE FUNCTION IN MYELOBLASTIC LEUKAEMIA

P. M. WILKINSON,* C. SUMNER, I. W. DELAMORE, C. G. GEARY AND G. R. MILNER

*From the University Department of Haematology, Manchester Royal Infirmary*

Received 9 June 1975. Accepted 16 July 1975

**Summary.—A study of granulocyte function in myeloblastic leukaemia is reported. Function was assessed by the ability of peripheral blood granulocytes to ingest and kill *Candida albicans in vitro*. Depressed cidal activity was observed in 11 patients with smouldering leukaemia and in 19 patients with acute myeloid leukaemia. Cidal activity was lowest in the untreated acute disease; this improved during cyto-reduction therapy and was maintained when remission occurred. Leukaemic plasma depressed the function of control granulocytes; the possible role of a plasma " factor " is discussed.**

THE NATURAL history of acute leukaemia is characterized by the development of frequent and severe infections, and infection is a major cause of death (Hersch *et al.*, 1965). Various theories have been proposed to explain the increased incidence of infection. Hirschenberg (1939) was the first to report impaired phagocytosis in acute leukaemia, an observation that has since been confirmed by many workers.

The purpose of this study was to examine granulocyte function in myeloblastic leukaemia at various stages of the disease. The results are presented and the findings discussed.

### PATIENTS AND METHODS

*Patients and controls.*—All patients studied had an unequivocal diagnosis of myeloblastic leukaemia on the basis of clinical, peripheral blood and bone marrow examination. The term acute myeloid leukaemia (AML) embraces all the morphological forms. Patients were investigated at presentation, during induction therapy and when in remission. Those receiving drug therapy had no chemotherapy for 5 days before the assay. Patients in remission were concurrently receiving immunotherapy with weekly injections of BCG and irradiated blast cells as part of the sixth MRC trial, but had received no therapy for 7 days before the assay.

The term " smouldering leukaemia " (SL) (Drayfus, Sultan and Manonni, 1970) was applied to those patients with abnormalities of the peripheral blood and/or bone marrow in which the percentage of blast cells in the marrow, whilst similar in morphology, was substantially less than in AML and did not account for more than 30% of the total marrow cells. Most of these patients were anaemic, thrombocytopenic and many granulocytopenic. Two patients were receiving daily prednisolone and blood transfusions when indicated. The remainder were not receiving chemotherapy or immunotherapy.

Control samples were obtained from healthy hospital personnel.

The effect of drug therapy on granulocyte function was assessed by incubating separately control cells with hydrocortisone (30 μg/ml), daunorubicin (3 μg/ml) and cytosine arabinoside (0·6 μg/ml).

Student's " *t* " test was employed to assess differences between the mean scores.

*Assay.*—The technique for assay was that described by Lehrer and Cline (1969) as modified by Goldman and Th'ng (1973).

*Candida albicans* was maintained on glucose–peptone agar at room temperature. Only cultures containing more than 95% of viable cells were used for the assay. Equal volumes (0·25 ml) of leucocyte suspension,

* Present address: Consultant in Clinical Pharmacology, Christie Hospital and Holt Radium Institute, Manchester.

autologous plasma and Hanks' balanced salt solution (HBSS) were placed in sterile containers and a suspension of *Candida albicans* added to provide a neutrophil : Candida ratio of 1 : 1. Each estimation was performed with control leucocytes and a blank tube containing plasma HBSS and *Candida albicans*, but no leucocytes.

Tubes were incubated at 37°C for 1 h. After 30 min a small sample was withdrawn to confirm that all the added organisms had been ingested. At 1 h Na deoxycholate (pH 8·7) was added to lyse the leucocytes and 5 min later methylene blue (0·25%) was added and the suspension centrifuged. From a wet preparation of each cell sediment 500 Candida cells were counted (dead cells stain a uniform intense blue). The Candidacidal index (%) was calculated as follows:

$$\frac{\text{Total number dead cells} - \text{number dead in control tube}}{5}.$$

### RESULTS

Sixteen control subjects and 30 patients with leukaemia were studied. Nineteen patients had AML (7 untreated, 6 were receiving drug therapy and 6 immunotherapy) and 11 patients smouldering leukaemia.

The Candidacidal index in control subjects was 12·4 ± 2·4% (range 10–18) and was highly reproducible.

The values obtained in granulocytic leukaemia are shown in Table I. Patients with both smouldering leukaemia and AML had a significantly reduced index compared with control subjects. The index was most impaired in patients with untreated AML (3·1 ± 0·6%); but improved during induction therapy (6·6 ± 1·5%), and later when in remission (8·7 ± 1·1%). The difference in Candidacidal index between the 4 groups was statistically significant (Table II).

The effects of leukaemic plasma on normal granulocyte function are shown in Table III. In all groups, the incubation of normal granulocytes with leukaemic plasma significantly depressed the index. In patients with untreated AML, the addition of normal plasma to leukaemic cells significantly increased the index.

Cytosine arabinoside and hydrocortisone did not depress the Candidacidal index in control subjects. The index fell to 8% when cells were incubated with daunorubicin.

### DISCUSSION

Various *in vitro* techniques are available to assess granulocyte function. The method used in this study was chosen because of the reproducibility of the test and because Candidiasis is the most

TABLE I.—*Candidacidal Index (%) in 30 Patients with Granulocytic Leukaemia*

| Disease | No. | Candidacidal index: Mean ± s.d. | Candidacidal index: Range |
|---|---|---|---|
| SL | 11 | 7·16±0·75* | 6·4–8·0 |
| AML (new) | 7 | 3·1±1·6† | 0–4·8 |
| AML (induction) | 6 | 6·6±1·46* | 4–8 |
| AML (immunotherapy) | 6 | 8·7±1·1* | 8–10·6 |

Values were compared with control subjects; an asterisk indicates a significant difference.
* $P < 0·05$, † $P < 0·01$ two-tailed. (For normal values see text.)

TABLE II.—*Difference in Candidacidal Index (%) in Smouldering and Acute Myeloid Leukaemia*

| Group | Difference | Group | Difference |
|---|---|---|---|
| SL *vs* AML (new) | 4·0† | AML (new) *vs* AML (induction) | 3·5* |
| SL *vs* AML (induction) | 0·6 | AML (new) *vs* AML (immunotherapy) | 5·6† |
| SL *vs* AML (immunotherapy) | 1·5* | AML (induction) *vs* AML (immunotherapy) | 2·1* |

* $P < 0·01$ two-tailed, † $P < 0·001$ two-tailed.

TABLE III.—*Candidacidal Index in Granulocytic Leukaemia; Effect of Leukaemic Plasma on Control Granulocytes and Control plasma on Leukaemic Granulocytes*

| | | Candidacidal index % | | | | | |
|---|---|---|---|---|---|---|---|
| | | Leukaemic cells | | | Control cells | | |
| Group | No. | Autologous plasma | Control plasma | Difference | Autologous plasma | Leukaemic plasma | Difference |
| SL | 11 | 7·2±0·75 | 7·6±0·78 | 0·4 | 10·5±0·9 | 9·2±0·8 | 1·3* |
| AML (new) | 7 | 3·1±1·6 | 5·2±3·6 | 2·1* | 11·1±2·3 | 8·1±0·8 | 3·0* |
| AML (induction) | 6 | 6·6±1·4 | 8·5±2·7 | 1·9 | 11·8±1·2 | 8·8±1·6 | 3·0* |
| AML (immunotherapy) | 6 | 8·7±1·1 | 9·4±1·0 | 0·7 | 12·0±1·6 | 10·3±2·1 | 1·7* |

$^*P = 0\cdot05$ two-tailed.

frequent fungal complication in leukaemia (Bodey, 1966). Because of the ratio of cells to organisms employed, the test is of value in assessing cidal but not phagocytic properties of granulocytes.

Granulocyte function in leukaemia has been studied extensively. The observation that granulocytes obtained from patients with AML have an impaired ability to kill *Candida albicans* confirms the results of previous workers (Lehrer and Cline, 1971; Goldman and Th'ng, 1973). There is, however, little work on granulocyte function in smouldering leukaemia, though these patients also show a high incidence of infection. In these patients, specific therapy is not normally indicated during the " smouldering " phase of the disease and transfusion requirements are often minimal. Bacterial infections, however, are common and impaired bactericidal activity has been demonstrated *in vitro* (Lehrer *et al.*, 1973). This study provides further evidence of defective granulocyte function. The functional capacity of the granulocyte in SL is greater than that observed in the acute phase of the disease. The Candidacidal index in the 2 patients in whom the acute phase was imminent fell to that observed in untreated AML, suggesting that there is a gradual impairment of function as the disease progresses.

In AML granulocyte function improved significantly during induction therapy (Table II) and this was maintained when remission occurred (Table I). The index did not return to the normal range, implying that the cell is still functionally abnormal and may support the view that granulocytes in AML in remission are derived from persistently abnormal stem cells. Hudson and Wilson (1960) also observed that leucocytes from patients in remission had decreased bactericidal activity. This functional abnormality may be due to persistent enzyme defects within the cell; such defects have been observed by other workers (Skeel, Yankee and Henderson, 1971; Strauss *et al.*, 1970).

Other factors not specific to leukaemia may depress the Candidacidal index, *e.g.* iron deficiency (Chandra, 1973) and abnormal immunoglobulin status (de Cree *et al.*, 1974). There was no evidence of iron deficiency in the patients studied and in most instances immunoglobulin levels were within the normal range. Complement levels were not assessed but Fahey and Boggs (1960) found no consistent abnormality in complement levels in acute leukaemia. The presence of antibodies against *Candida albicans* would not produce a low index as the anti-Candida antibody does not kill or lyse Candida organisms *in vitro* (Lehrer and Cline, 1969). We found no evidence that blast cells or immature granulocytes had significant cidal activity, confirming the observations of other workers (Silver *et al.*, 1957; Lichtman and Weed, 1972). While daunorubicin was found to depress normal granulocyte function, because of the interval between administration and performing the assay (10 days) the drug residue remaining in the body was negligible and unlikely to invalidate the assay. Thus, the observed defect in granulocyte function must reflect an intrinsic abnormality.

While previous studies have generally been concerned with the granulocyte, little attention has been paid to extrinsic factors. The effects of leukaemic plasma on normal granulocyte function was first noted by Braude, Feltes and Brooks (1954) and later by Goldman and Th'ng (1973).

Plasma from patients with acute AML depressed the cidal capacity of normal granulocytes. The explanation of this phenomenon must remain a matter for conjecture for the present. There is some evidence to suggest that it be due in part to a factor released from the leukaemic blast cell. The Candidacidal index in patients with untreated AML improved when granulocytes were incubated with control plasma (Table III) but not when remission occurred. There was no evidence that granulocyte function in smouldering leukaemia was related to the number of blast cells in the blood. Mir and Bobinski (1975) observed that a blast cell extract impaired the sodium " pump " of normal red blood corpuscles. These observations suggest the presence of an enzyme inhibitor, probably a protein, which may have protean effects on cell function.

Leukaemic plasma from patients with AML in remission and with smouldering leukaemia also depressed normal function. Patients in remission had received several blood transfusions and weekly injections of BCG and allogeneic blast cells; the resulting antibody production may have impaired normal function. Patients with smouldering leukaemia, however, were receiving neither blood transfusions nor specific drug therapy. The ability of plasma to depress normal function in these patients may be due to the development of an abnormal protein which further impairs granulocyte function when the acute stage develops.

### REFERENCES

Bodey, G. P. (1966) Fungal Infections Complicating Acute Leukaemia. *J. chron. Dis.*, **19**, 667.

Braude, A. I., Feltes, J. & Brooks, M. (1954) Differences between the Activities of Mature Granulocytes in Leukaemia and Normal Blood. *J. clin. Invest.*, **33**, 1036.

Chandra, R. Y. (1973) Reduced Bactericidal Capacity of Polymorphs in Iron Deficiency. *Archs dis. Childh.*, **48**, 864.

de Cree, J., Verhaegen, H., de Cock, W. *et al.* (1974) Impaired Neutrophil Phagocytosis. *Lancet*, ii, 294.

Drayfus, B., Sultan, C. & Manonni, P. (1970) Refractory Anaemia with Excess of Myeloblasts in the Bone Marrow. A Study of Eleven Cases. Combined Meeting of the French and British Societies for Haematology. Cambridge, April 1970. Abstract *Br. J. Haemat.*, 1971, **20**, 669.

Fahey, J. L. & Boggs, D. R. (1960) Serum Protein Changes in Malignant Disease. The Acute Leukemias. *Blood*, **16**, 1479.

Goldman, J. M. & Th'ng, K. H. (1973) Phagocytic Function of Leucocytes from Patients with Acute Myeloid and Chronic Granulocytic Leukaemia. *Br. J. Haemat.*, **25**, 299.

Hersch, E. M., Bodey, G. P., Nies, B. A. & Freireich, E. J. (1965) Causes of Death in Acute Leukemia. *J. Am. med. Ass.*, **193**, 105.

Hirschenberg, N. (1939) Phagocytic Activity in Leukemia. *Am. J. med. Sci.*, **197**, 706.

Hudson, R. P. & Wilson, S. J. (1960) Hypogammaglinemia and Chronic Lymphatic Leukemia. *Cancer, N.Y.*, **13**, 200.

Lehrer, R. I. & Cline, M. J. (1969) Interaction of *Candida albicans* with Human Leucocytes and Serum. *J. Bact.*, **98**, 996.

Lehrer, R. I. & Cline, M. J. (1971) Leukocyte Candidacidal Activity and Resistance to Systemic Candidiasis in Patients with Cancer. *Cancer, N.Y.*, **27**, 1211.

Lehrer, R. I., Goldberg, L. S., Apple, M. A. & Rosenthal, N. P. (1973) Refractory Megaloblastic Anemia with Myeloperoxidase Deficient Neutrophils. *Ann. intern. Med.*, **76**, 447.

Lichtman, M. A. & Weed, R. I. (1972) Alteration of the Cell Periphery during Granulocyte Maturation: Relationship to Cell Function. *Blood*, **39**, 301.

Mir, M. A. & Bobinski, H. (1975) Altered Membrane Sodium Transport and the Presence of a Plasma Oubain-like Inhibitory Factor in Acute Myeloid Leukaemia. *Clin. Sci. molec. Med.*, **48**, 213.

Silver, R. T., Beal, G. A., Schneiderman, M. A. & McCullough, N. B. (1957) The Role of the Mature Neutrophil in Bacterial Infections in Acute Leukemia. *Blood*, **12**, 814.

Skeel, R. T., Yankee, R. A. & Henderson, E. S. (1971) Hexose Monophosphate Shunt Activity of Circulating Phagocytes in Acute Lymphocytic Leukemia. *J. Lab. clin. Med.*, **77**, 975.

Strauss, R. R., Paul, R. B., Jacobs, A. A., Simmons, C. & Sbarra, A. J. (1970) The Metabolic Phagocytic Activities of Leukocytes from Children with Acute Leukemia. *Cancer Res.*, **30**, 480.